# COVID-19: A systematic evaluation of personal protective equipment (PPE) performance during restraint

**DOI:** 10.1177/00258024211000805

**Published:** 2021-03-09

**Authors:** Roland Dix, David Straiton, Peter Metherall, James Laidlaw, Lisa McLean, Andy Hayward, Gary Ginger, Louise Forrester, Paul O’Rourke, Rob Jefferies

**Affiliations:** 1 Gloucestershire Health and Care NHS Foundation Trust, Montpellier Unit, Wotton Lawn Hospital, UK; 2 Sheffield Teaching Hospitals NHS Foundation Trust, Northern General Hospital, UK; 3 Avon and Wiltshire Mental Health Partnership NHS Trust, UK

**Keywords:** COVID-19, infection control, mental health, personal protective equipment, physical intervention, restraint

## Abstract

**Background:**

Restraint is widely practised within inpatient mental health services and is considered a higher-risk procedure for patients and staff. There is a sparsity of evidence in respect of the efficacy of personal protective equipment (PPE) used during restraint for reducing risk of infection.

**Methods:**

A series of choreographed restraint episodes were used to simulate contact contamination in research participants playing the roles of staff members and a patient. For comparison, one episode of simulated recording of physical observations was taken. Ultraviolet (UV) fluorescent material was used to track the simulated contact contamination, with analysis undertaken using established image registration techniques of UV photographs. This was repeated for three separate sets of PPE.

**Results:**

All three PPE sets showed similar performance in protecting against contamination transfer. For teams not utilising coveralls, this was dependent upon effective cleansing as part of doffing. There were similar patterns of contamination for restraint team members assigned to specific roles, with hands and upper torso appearing to be higher-risk areas. The restraint-related contamination was 23 times higher than that observed for physical observations.

**Discussion:**

A second layer of clothing that can be removed showed efficacy in reducing contact contamination. PPE fit to individual is important. Post-restraint cleansing procedures are currently inadequate, with new procedures for face and neck cleansing required. These findings leave scope for staff to potentially improve their appearance when donning PPE and engaging with distressed patients.

## Introduction

The COVID-19 pandemic creates challenges for infection control in mental health inpatient units. One particular concern is the risk of infection when engaged in physically restraining a patient, often referred to in mental health practice as ‘physical intervention’ (PI). PI is a difficult area of practice which can be very distressing for patients and staff, with significant risks of injury. There are national programmes for reducing the need for PI.

At times PI is necessary to contain serious risks arising from acutely disturbed behaviour. Within the UK, there were around 60,000 episodes of restraint reported in mental health services between 2016 and 2017.^1^

Preliminary review of the literature indicates that there is a sparsity of evidence in respect of personal protective equipment (PPE) used during PI in mental health settings. The majority of higher-quality evidence for PPE used in health care is focused upon the effective use of PPE in general medical hospitals (GMH).^2–5^

This paper describes a systematic evaluation of PPE undertaken to improve the understanding of the performance of PPE during episodes of PI. The project design was developed by the Gloucestershire Health and Care NHS Foundation Trust in collaboration with the National Association of Psychiatric Intensive Care Units (NAPICU), 3D Imaging Lab, Sheffield Teaching Hospital NHS Trust and Avon Wiltshire Partnership NHS Trust.

### Current mental health PPE guidance background

Public Health England (PHE) has issued guidance for PPE use in mental health settings.^6^ However, no specific additional recommendations are made for the mental health procedure of PI.

NAPICU published guidance on managing acute disturbance in mental health which included recommendations for PPE in PI.^7^ These recommendations were based on basic tests of PPE undertaken by PI training teams. The tests primarily considered which PPE was most likely to remain in place during PI.

At the time of writing, high-quality evidence or evaluation of PPE effectiveness in PI procedures within the mental health setting is absent. This contrasts with general medical settings where specific types of PPE have been evaluated for use in specific procedures, e.g. aerosol-generating procedures.

The PPE currently specified by PHE for use in mental health inpatient settings is focused upon carrying out procedures that would also be carried out in a GMH setting. Examples of this include procedures which require staff to be within 2 m or in contact with a patient, such as assistance with activities of daily living and recording physical observations.

There are procedures carried out in mental health settings that are comparable to general medical settings which require airway support, for example, resuscitation and electro-convulsive therapy, with the latter requiring an anaesthetic. For these procedures, current evidence supporting use of specific PPE is considered largely applicable to mental health settings.

### PPE-relevant procedures specific to mental health inpatient settings

Providers of mental health inpatient services, particularly ‘locked door services’, have an authorised approach to managing disturbed/aggressive behaviour which can extend to PI. For the UK, a broad description of PI is contained within Chapter 26 of the Mental Health Act Code of Practice (2015).^8^

### Physical intervention

There are a variety of different systems for PI used by mental health service providers in the UK, although all share similar characteristics. These are:

- A team of staff (often three) with specific roles, i.e. number one responsible for supporting the head and numbers two and three assigned to securing of the patient’s arms;
- A defined series of techniques that can be employed by members of staff to physically intervene to restrict the movement and contain serious risk represented by the behaviour of a patient;
- PI for relocating a person presenting various levels of resistance;
- PI methods for containment of risk within a de-escalation process resulting in the discontinuing of the need for PI;
- PI within which it is possible to administer medication parenterally.

### Physical intervention and risk

PI carries its own risks of injury to patients and staff. These include uncertainty as to the level of control that can be achieved in any given situation. Furthermore, PI episodes can often be difficult to predict in terms of the levels of resistance, amount and nature of close contact, or the length of time an episode will take to conclude.

PI is considered amongst the highest-risk procedures used in mental health settings and is governed by law. It often causes distress and should only be implemented when there are no less restrictive alternatives. Reducing risk of injury for all those involved is a central international consideration for the application of PI.^9,10^

It has long been recognised that at times additional risks to the staff can include increased possibility of infection from close proximity spitting, biting and scratching.^11,12^

### Physical intervention and risk to staff of COVID-19 infection

Since first being identified in December 2019, COVID-19 has proven to be highly infectious, leading to unprecedented measures of ‘lockdown’ and mandatory social distancing. Specific details of how COVID-19 spreads remain subject to study. It has been established that contact contamination represents a significant infection method. The extent to which COVID-19 can spread by aerosol remains subject to debate.^13^

Episodes of PI require very close physical contact between staff and patient. Within the process of close contact, there is also potential for physically challenging struggle during which opportunities for contact transmission of COVID-19 are increased.

The following are common characteristics of PI which may be considered to increase the risk of COVID-19 and other infections:

- Bodies are in physical contact with each other, particularly hands, providing direct opportunity for contact contamination.
- The extent to which any PPE will remain in place and able to withstand high levels of demanding physical activity.
- The potential for any PPE to be purposely damaged or attempts at removal made by the recipient of PI.
- Potential for very close proximity between the head/oral and nasal region between those involved in PI. This can range from 50 mm to 500 mm.
- Potential for very close-proximity directed projection of larger droplets of oral fluid, e.g. by spitting.
- Potential for very close-proximity shouting, coughing and raised voice projecting smaller particles of respiratory and oral secretions.^14^
- Increased respiratory rate and depth resulting from physical exertion, increasing the possibility of secretion and/or inhalation of virus-containing material.

### Evaluation questions

This paper aims to address the following questions:

- How does PPE perform in mitigating contact contamination during PI?
- What is the pattern of contact contamination arising from PI?
- What are the specific PI issues for the robustness and comfort of PPE?

This paper aims also to identify suggestions for improvement.

## Methods

### Review of literature

We searched nine databases including BNI, PsycInfo, Cinahl, Embase, Medline, Emcare, Google Scholar, World Health Organization, Global research on Coronavirus disease (COVID-19) database and medRxiv/bioRxiv COVID-19 SARS-CoV-2 preprints. We searched for all published evidence within these databases before 6 May 2020. We used search terms of Coronavirus, COVID, infection, infection control and physical restraint, physical intervention, restraint, mental health, psychiatry. In total, 276 published papers were retrieved; however, none contained evidence specifically covering PPE and infection control during restraint in mental health.

### Contact contamination

Twelve participants, divided into three groups of four, were used to undertake three episodes of simulated PI. Each of the episodes involved one participant representing the infection source (patient) and the remaining three representing members of a PI team (staff). In each episode the simulated PI followed a predetermined choreography, and in each episode the group playing the role of staff donned different PPE.

The participant representing a patient infected with COVID-19 had ultraviolet (UV) material placed in the areas of the body most likely to contain infectious material, i.e. lower face, upper chest, arms and hands. Spitting of oral fluid was simulated using UV fluorescent material consistent with training aids for infection control. All three episodes were completed using different staff in physically separate areas to remove the possibility of cross-contamination between the three episodes.

Following the PI episode, the amount and location of contact transfer of UV fluid between simulated patient and staff was recorded by UV photography.

### Non-contact contamination characteristics of PPE performance

The following was also subject to evaluation:

1. The extent to which the PPE remained in place.
2. Potential or actual hazards arising from the PPE.
3. The ease with which the different PPE sets could be donned and doffed.
4. Comfort of the PPE during use.

### Criteria for selection of PPE sets evaluated

In the PI scenario, the extent to which PPE will remain in place may be equally as important as the infection control specification.^7^ Pre-evaluation tests determined that the following criteria for the selection of the PPE should be formally evaluated:

- PPE generally considered mainstream with commercial mass production.
- PPE items were likely to remain in place.
- PPE items less likely to increase risk in the context of PI (e.g. slip hazard).
- PPE items known to have some infection control value.
- PPE worn as standard by staff during shifts.

The items were likely to have differences in the PI context worthy of comparison. These included:

F. Efficacy in preventing transfer of simulated infectious material.
G. Time taken to don and doff.
H. Potential for cross-contamination while doffing.

### Infection source test subject (ISTS) preparation

Under the supervision of an Infection Control Specialist Nurse (ICSN), the same amount of UV fluorescent substance was placed on three different participants assigned the role of patient on the areas likely to contain infectious fluid on a COVID-19-positive patient. These areas were hands, nasal and face region, and the anterior aspect of patient torso. The face and arms were contaminated using two different colours (hands and arms green coloured, face and torso blue coloured).

At two points during the PI episodes spitting was simulated. This was achieved by means of a spray bottle containing UV material of a third colour (red). This was directed towards a participant’s face to simulate transfer of oral fluid by spitting.

- Point One: spray bottle 150 mm away from the target.
- Point Two: spray bottle 300 mm away from the target.

### Test staff members (TSM) preparation

#### Pre-test donning and doffing preparation

**Figure 1.**
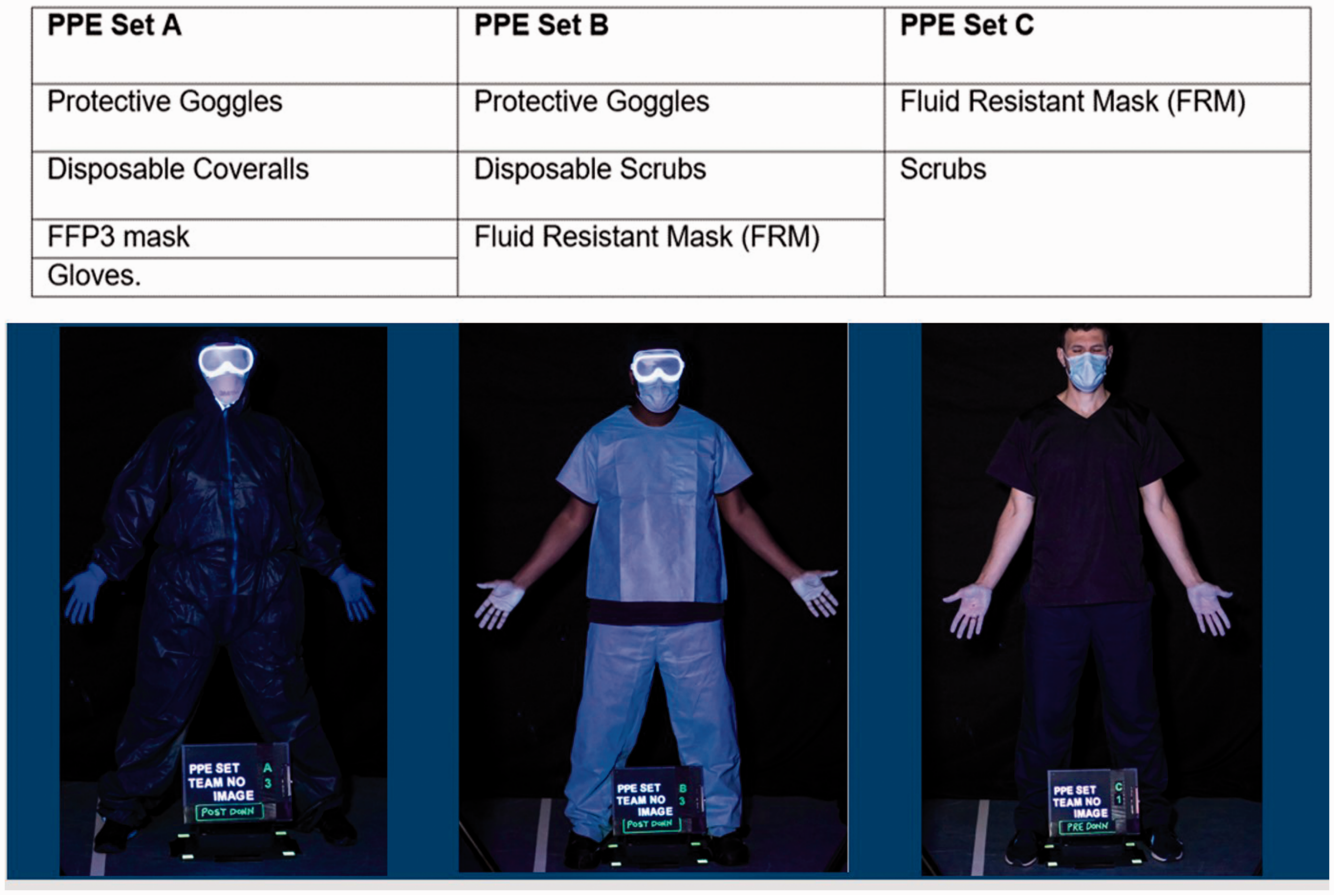
PPE sets donned.

After standard training following an Infection Control Action Card, nine participants who were staff from the Physical Interventions training team assigned to the role of TSM (staff members) donned and doffed the three different PPE sets in Figure 1.

#### Donning

Donning and doffing for all TSMs took place in an area of 4 square metres with two chairs present and a standard size peddle bin. Each team of three TSMs had a separate area. Time taken to don was recorded. Donning and doffing was observed by the ICSN, who recorded any issues against a standard checklist. Any correction advice needed or offered by the ICSN was also recorded.

#### Cleansing

In order to simulate the cleaning facilities available at the site of PI episodes in mental health inpatient facilities, universal wipes (branded Clinell) were used for personal cleansing.

#### PI simulation

The following PI procedures were choreographed and enacted in three episodes as a linear progressive sequence of similar time duration (4.5 min).

- Immediate containment of assault;
- Relocation;
- De-escalation.

### Data collection and analysis

#### Contact contamination

A darkened photograph booth was constructed which was illuminated with visible and/or UV light. Within controlled parameters, full-body photographs were taken of the test subjects at the following points:

1. Pre-donning PPE;
2. Post-donning PPE;
3. Post-PI simulation with PPE;
4. Post-doffing without PPE.

PPE Set C did not require donning of PPE as they were already wearing scrubs and a mask, representing the standard uniform of a member of staff on an inpatient mental health ward. As such, there were only three sets of photos required for this group.

#### Image analysis

Comparison of the simulated contamination was made between the different PI episodes and contamination types using a standardised analysis of the UV photographs. Established deformable image registration techniques were implemented (MIM Software, Cleveland OH) to align all visible light photographs for the definition of nine standard regions (head, neck, four thorax regions, arms, hands and legs). These were then applied to the UV photographs to measure the regional distribution of the contamination.

Detection of the coloured dyes was performed by segmenting the UV photographs using a clustering methodology (*k*-means clustering with UV images converted to the CIE L*a*b* colour space).^15^ Here the dominant colour of each dye (and other background features) was found from photographs of the ISTS prior to the PI. These colours were then used to partition the TSM photographs to identify areas of contamination. Combining this segmentation with the regional body contours permits a zonal analysis. Some manual editing was required to exclude regions of clothing which appeared blue in the UV image.

#### Non-contact contamination characteristics of PPE performance

Each TSM was observed during and interviewed following the three simulations. The three episodes were video recorded (including donning and doffing). The videos were reviewed by a panel of Infection Control and PI specialists to identify any issues arising.

## Results

### Determining the extent of contamination

**Figure 2.**
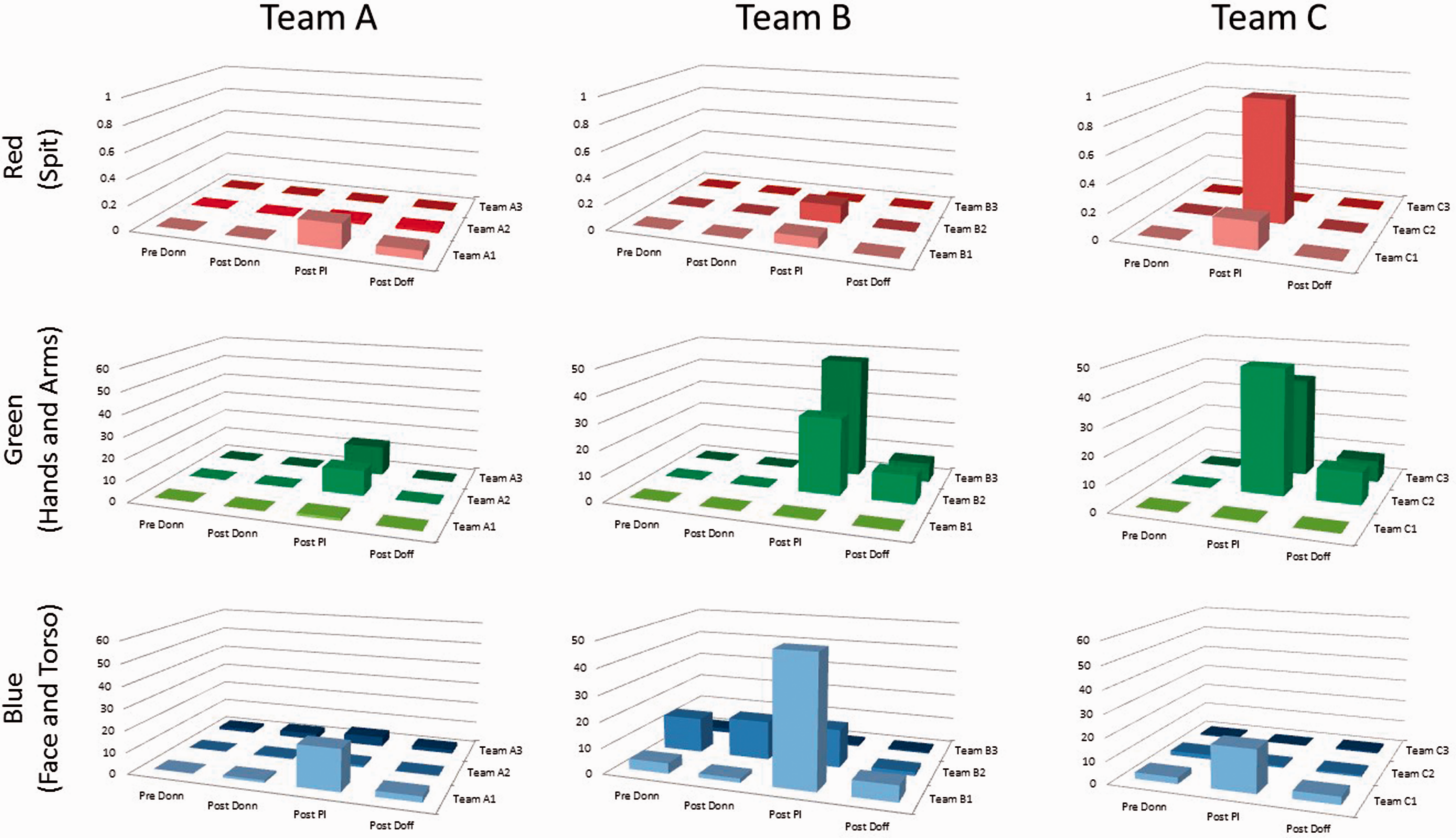
Graph to show the origin and total accumulation of UV material (‘contamination’) during the sequential experiment steps.

There was evidence of contact transfer during the simulated episodes of PI (Figure 2).

For general reference, a participant not involved in the PI episodes was ‘contaminated’ in the same manner as the simulated PI patient with UV substance. Following this their general physical observations (blood pressure, temperature and O_2_ saturation) were taken. The same method for tracking contact contamination was applied. When compared with taking physical observations, 23 times more contact contamination occurred following an episode of PI (single observation subject compared with average of all TSM participants).

Figure 2 shows a series of graphs demonstrating the origin and total surface area of UV material (representing contamination) for each team member during the sequential experiment stages; pre and post PPE donning, post-PI and post-doffing. Area units are arbitrary as no calibration of the camera system was performed; however, the results can be compared between subjects owing to the strict experimental method.

In all cases the graphs show peak contamination post-PI, but there are several cases where the contamination persists post-doffing. For the face and torso contaminate (blue dye), there is some level of UV fluorescence similar to contamination detected prior to donning of the PPE. This was considered likely to have originated from the Clinell wipes used to clean prior to donning.

**Figure 3.**
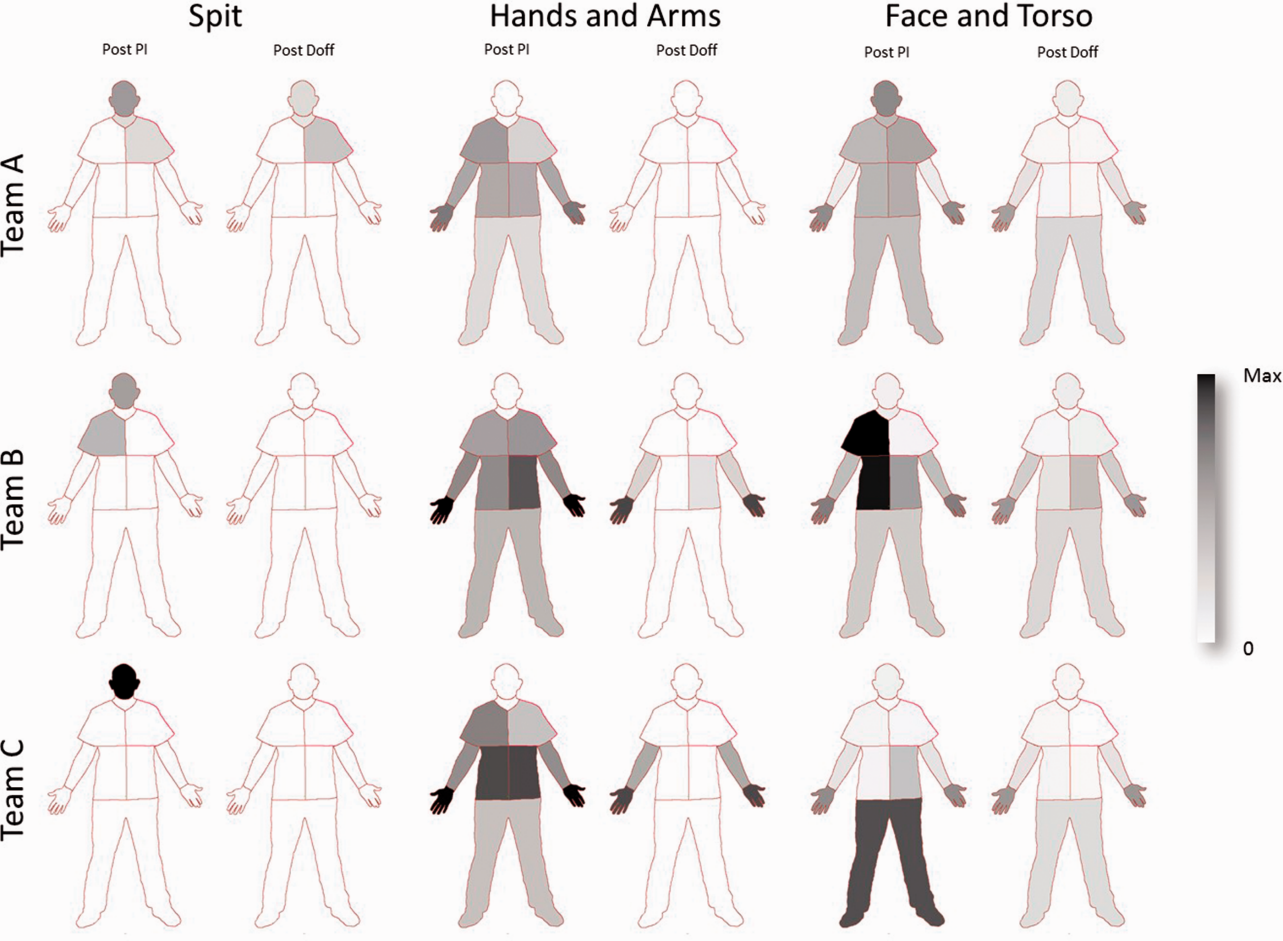
Regional contamination distribution. Comparison of post-PI to post-doff for each team. The totalised contamination for each team is shown normalised to the maximum in all three teams.

Using the standardised regions, the regional distribution of contamination is shown for each team in the schematic representation of Figure 3. A graded greyscale has been used to display the summed contamination for each team, normalised to the maximum level found in all three teams. Each contamination type is treated separately.

The spit contamination (red) is concentrated around the head for all groups, although for team A and B some was also deposited on the upper torso. Team A showed some contamination remaining post-doffing.

The contamination derived from the hands and arms (green) of the patient was spread much more widely, but none was detected on the face and neck for any team. Team A, post-doffing had virtually no contamination, with only a very small amount remaining on the hands of subject A2. Teams B and C, however, show contamination remaining post-doffing, and this is concentrated on the hands and to a lesser extent on the arms and lower torso (Team B).

The contamination from face and torso (blue) showed a wider distribution including the head and neck. Similarly, the post-doff distribution was also wide, with the highest level found in the hands, but for team B contamination was still detected on the face. In all teams it was found that the PPE and skin-cleaning product was clearly visible in the UV images, which in some cases was difficult to differentiate from the contaminant dye.

**Figure 4.**
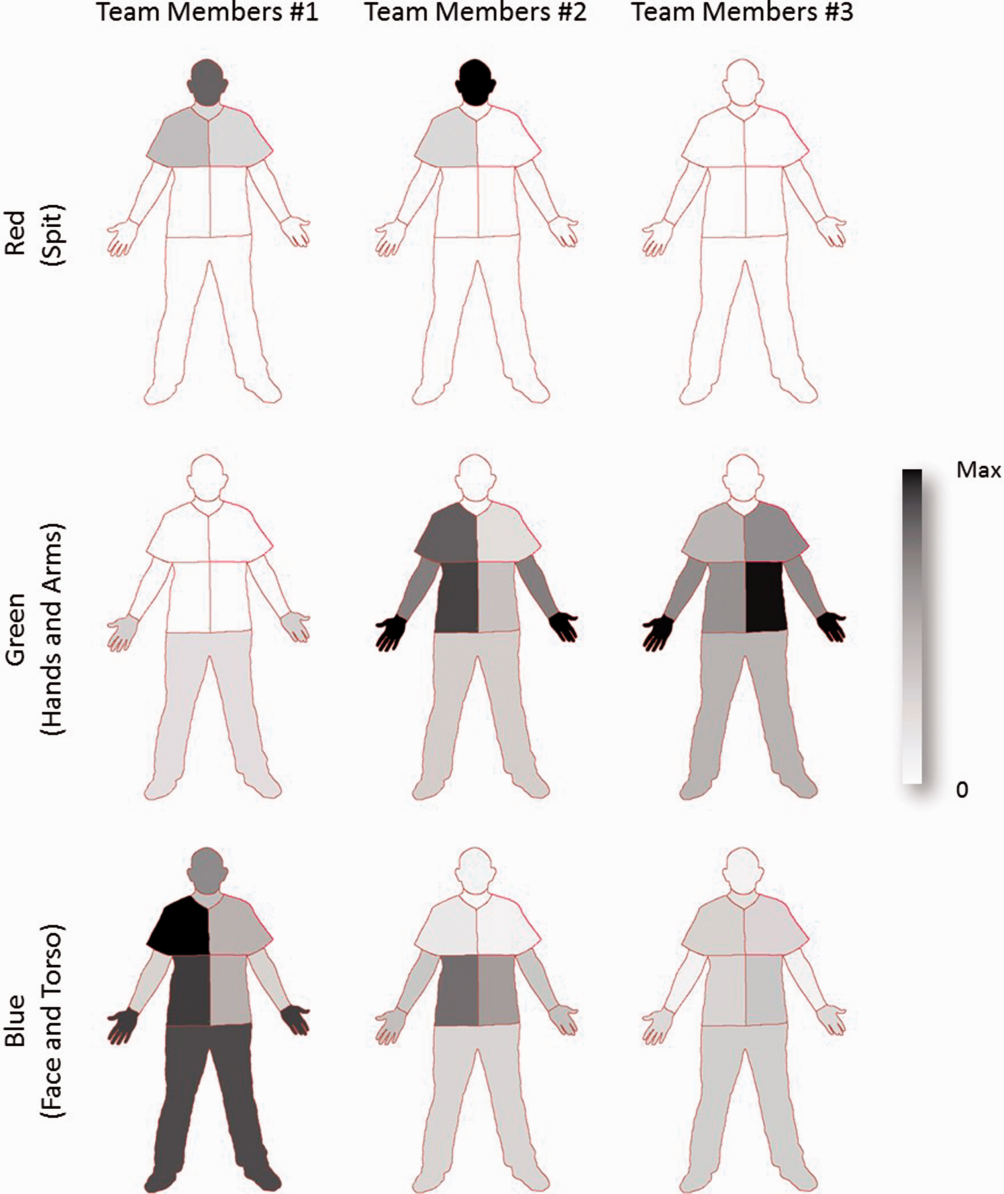
Regional contamination distribution, comparison of aggregate post-PI for team members one, two and three. The totalised contamination for each team member is shown normalised to the maximum for each contamination type.

By aggregating the post-PI results for team members one, two and three over all the three groups, it is possible to see where the contamination is predominately derived from (Figure 4). The red (spit) contamination was only detected on team members one and two, and this was concentrated around the head with a smaller amount seen on the upper torso. The green contamination, originating from the hands and arms of the patient, shows that this was spread mainly to the hands and to the torso. The distribution for team members two appears to be roughly a mirror image of team members three. The blue contamination appears to spread much more widely, with no clear pattern demonstrated.

**Figure 5.**
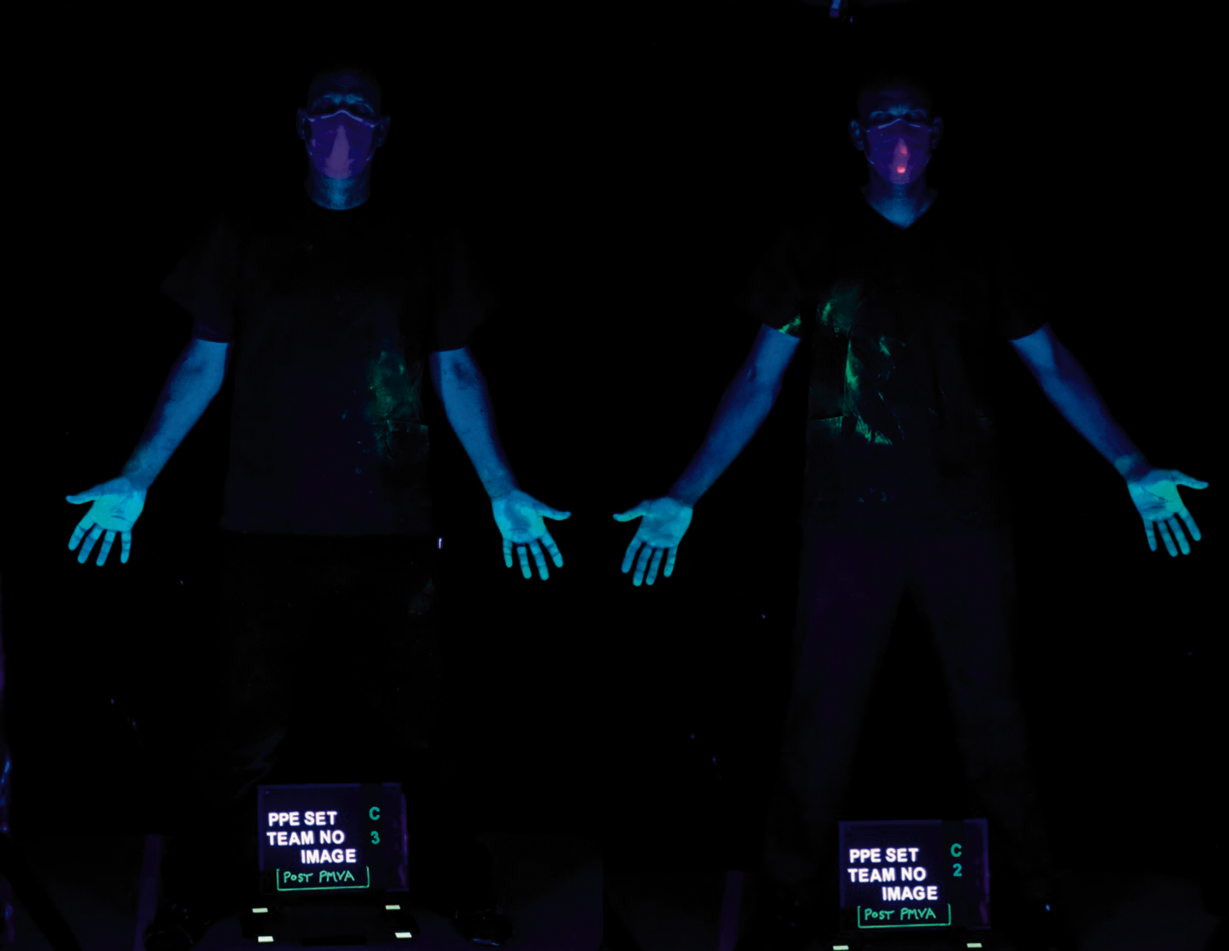
Post-PI contamination of PPE Team C members 2 and 3.

**Figure 6.**
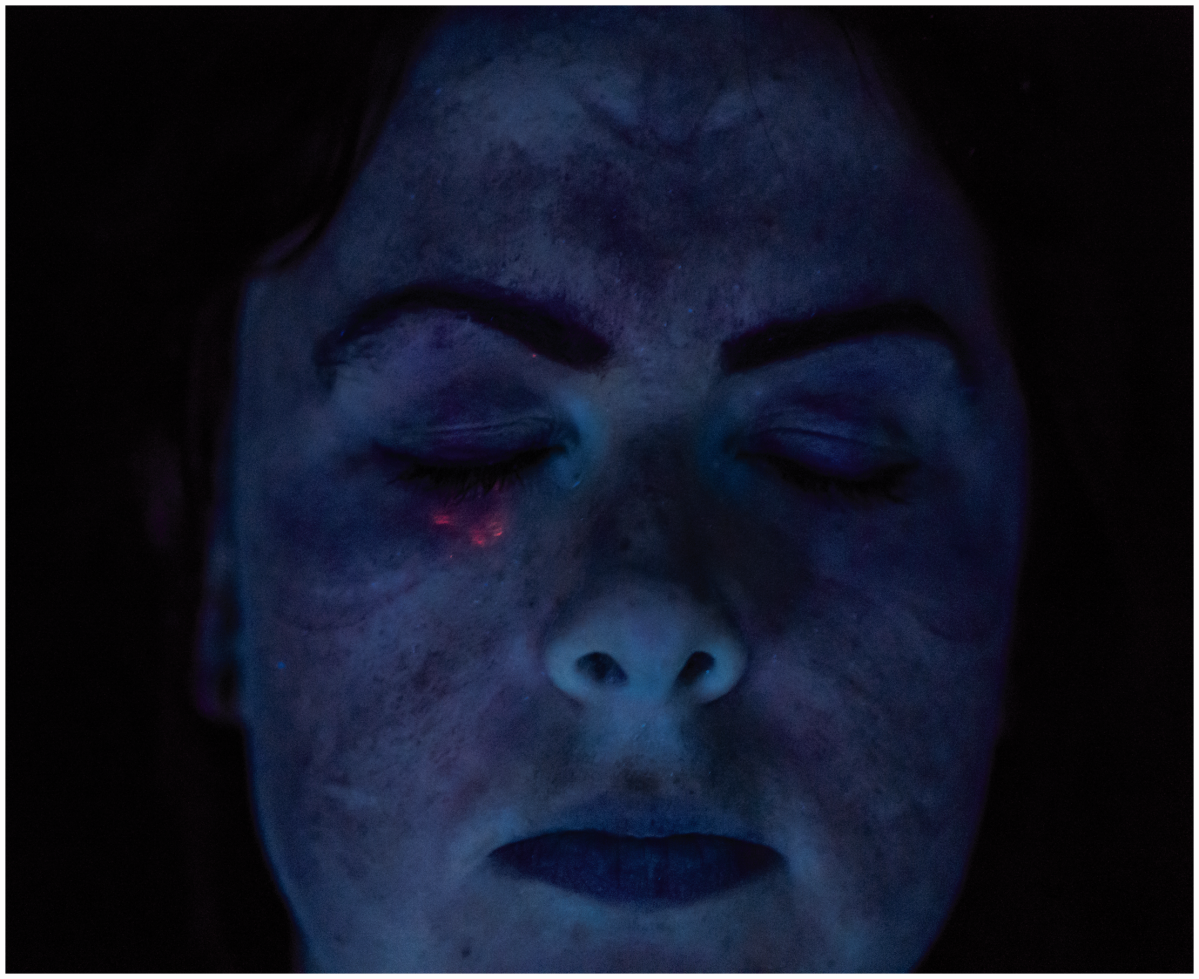
Post-PI contamination of PPE Team A member 1.

### Summary of results

All three PPE sets showed similar performance in protecting against contamination transfer. For teams not utilising coveralls, this was dependent upon effective cleansing as part of doffing. There were similar patterns of contamination for restraint team members assigned to specific roles, with hands and upper torso appearing to be higher-risk areas. The restraint-related contamination was, on average, 23 times higher than that observed for the recording of physical observations.

### Non-contact contamination characteristics of PPE performance

**Goggles:** The goggles remained in place, but on one occasion there was evidence of UV material breaching the goggles. The goggles quickly misted up and severely restricted vision. TSM reported some discomfort after 20 min.

**Masks:** There were no issues with FFP3 and fluid-resistant masks in terms of comfort or hazards. They remained in place.

**Coveralls:** The zip marginally opening during the PI procedures caused high levels of heat and perspiration. TSM reported that the coveralls were very uncomfortable from overheating.

**Disposable scrubs:** There was one incident of the disposable scrubs ripping due to inappropriate sizing. However, they remained in place and were felt to be comfortable.

**Scrubs:** Remained in place, were comfortable, but had to replaced by alternative clothing post-doffing.

## Discussion

### Contact contamination

All three of the PPE sets showed similar performance in the amount of transfer of contaminant. Pre-doffing, the location of containment was surprisingly similar between the teams.

Only PPE set A (disposable coveralls) provided protection of the arms. This set showed less UV contaminant in contact with skin in the arms area post-PI compared with Set B and Set C.

Of particular note was contaminant located in axilla region (coloured green) of numbers two and three in the PI teams (Figure 5). This arose from contact with the infection source hands and arms when they were secured using PI techniques. Numbers two and three (left and right side of the patient) in the teams received less contamination (coloured blue) compared with team member one originating from the face and chest area of the infection source.

Number one in the PI team (responsible for the controlling the head) had more UV material than the other team members on their hands and wrist which transferred from the face and torso area (coloured blue) of the infection source. Number one in the teams received less contamination from the hands and arms of the infection source (coloured green) compared with the other two team members.

Following doffing, all three PPE sets showed similar performance in protecting against contamination transfer. For teams not utilising coveralls, this was dependent upon effective cleansing as part of doffing. One of the test subjects clearly missed cleansing an area of contact contamination in the arms. If the PPE had provided arm coverage, it is likely that this area would not have remained contaminated post-doffing.

### Spitting

The spitting simulation (coloured red) presented particular challenges for the PPE. Both the fluid-resistant and the FFP3 mask were successful in preventing contact contamination. However, spit contaminant did breach the goggles and was found on the lower eye area of one of the test subjects (Figure 6). Contaminant was also found on the neck area, and automatic analysis identified small regions over the torso in a number of subjects.

Careful review of the video recording could not clearly establish how the contaminant breached the goggles. Theories include the front panel of the goggles becoming dislodged from the frame or the face seal of the goggles becoming displaced during the PI episode.

Spitting demonstrated the clear need for face and neck cleansing procedures that currently do not feature in standard cleansing advice for health care.

### Non-contact contamination characteristics of PPE

This evaluation has demonstrated that PPE needs to be properly fitted to the wearer. Examples of this include the coverall zips travelling downwards during simulation as a result of being oversized for the individual. There was also an episode of disposable scrubs ripping on donning as a result of them being too small for the test subject’s legs.

Two areas of major concern include the overheating experienced by the test subjects from the coveralls, and the goggles misting up to the point of severely restricting the vision of the PI team members.

### Implications for clinical practice

It has been long established that donning and doffing PPE is crucial to its effective performance. This also requires PPE to be specifically fitted to the individual rather than limited to a small number of sizing options. This evaluation showed diminished performance for some poorly fitting PPE.

For mental health inpatient practice, face and neck cleansing procedures are required and are possibly more important than previously thought. These are required to deal with the risk of spitting or experiencing pressure of speech while unable to observe social distancing.

Debate continues as to the extent to which COVID-19 is airborne, although this was not the focus of this evaluation. Given the specific characteristics of PI (physical excretion, very close contact, elevated voices), FFP3 standard masks would likely be preferable to fluid-resistant masks.

The specific context of PI involving high levels of physical exertion is a central PPE consideration. This requires PPE to be robust, comfortable and well secured. Of specific note is the need for eye protection to be robust and include mitigation against misting. This could be achieved by selecting robust goggle designs and preparing them with an anti-mist spray.

While offering higher levels of protection, overheating caused by coveralls as well as more difficult doffing observed in this evaluation suggested that alternatives could return satisfactory performance while mitigating these problems.

The evaluation suggests that a second layer of clothing which can be removed can be effective in minimising contact contamination following PI. This is, however, dependent upon there being effective cleansing equipment and procedures available following PI. Exactly what the second layer is may be less relevant than its presence.

This could be an important consideration in selecting PPE, providing more options including those with improved appearance for patients who may already be anxious and frightened in the acute mental health context. The role of each PI team member within the procedure was shown to be relevant to the higher-risk areas for contact contamination. For the staff member in charge of controlling the head, the hand and wrist areas were at higher risk of contamination. For those assigned to each side of the patient, then the respective side of the axilla region as well as hands and wrist were higher-risk areas.

This evaluation demonstrates the need for further high-quality evidence which is derived from the unique characteristics of mental health inpatient practice. The simulated PI used in this study is specific to inpatient mental health services. However, our findings may also have relevance to other public service sectors such as the police, prison service, care home and residential settings where PI may be implemented.

### Ethical considerations

Members of the local ethics committee were consulted. The evaluation design did not involve any patients or patient-related data and therefore is considered part of standard procedure and equipment evaluation not requiring ethical approval. Valid consent to take part in the evaluation was provided by all involved.

### Limitations of evaluation

The evaluation was restricted to contact transfer of potential virus-containing material from the patient to the staff acting in the PI. The evaluation did not consider the potential for infection to be transferred from the staff to the patient in similar circumstances. Nevertheless, the conclusions drawn may guide support for patients in the areas of cleansing and changing clothes.

The evaluation did not consider potential for aerosol transmission of smaller material.

The transfer of UV material cannot be considered as an accurate representation of the volume of potentially virus-containing material transferred, although it does give an indication of the contact transmission areas that could contain virus material.

Some items of PPE (notably gloves and goggles) and the universal wipe used to clean the skin were clearly visualised in the UV light photographs with a colour close to the fluorescent dye used to represent contamination. This made automatic segmentation of the dye distribution difficult.

Regions of contamination appeared overexposed in the UV photographs, with a bright white appearance. This was difficult to distinguish from lower-level contamination of the blue fluorescent dye.

The high-resolution close-up photographs showed that some contamination was not detected in the 32 whole-body images, albeit only very small amounts.
